# From Strain toward Hyperdoppler Echocardiographic Evaluation in Sports Medicine

**DOI:** 10.3390/ijerph19137702

**Published:** 2022-06-23

**Authors:** Laura Stefani

**Affiliations:** Sport Medicine Center, Department of Experimental and Clinical Medicine, University of Florence, 50100 Florence, Italy; laura.stefani@unifi.it; Tel.: +39-3477689030

## 1. Introduction

Sports medicine is often involved in the evaluation of a wide population composed by active or less active individuals. The context of sports medicine assessment is recently moved from the high-level athletes toward general population. In many cases some specific aspects of the cardiac function need to be investigated in deep to exclude any eventual structural myocardial damage. 

Despite the first approach is usually the ECG exam, that is mandatory to obtain the eligibility, some additional evaluations are important. Among them, the echocardiographic exam represents the first non-invasive investigation after ECG evaluation. 

Trained athletes can show some “grey zone” of interpretation, due to cardiac morphological modifications induced by exercise [1]. The decision making of the sports eligibility can be sometimes difficult as consequence of the modifications of all the cardiac chambers due to exercise.

In addition to the normal population of athletes, the emerging classes of athletes, such as subjects with previous cancer or transplants story, need to be studied using specific cardiac echographic parameters, either in the exercise prescription process, a a structured phyical activity program, or in the eligibility.

Especially master athlete’s category can show various clinical condition, suggestive for coronary artery disease (CAD) or mild left ventricular hypertrophy (LVH), as well ST-T abnormalities particularly if associated with LBBB. Particularly this last ECG pattern, is important for sport medicine, as possible consequence of a structural cardiomyopathy, potentially related to an ischemic event. For this reason, master athletes, affected by LBBB are often investigated using echocardiography stress test. 

No data are now available about the effective impact of a regular sports activity in them, and an absolute contraindication does not exist among the principal recommendations.

Another dubious condition is represented by QRS ECG fragmentation, which could be expression of a pathological substrate [2]. It is not clear if this last ECG aspect, found in high trained athletes, age related, is connected to a pathology or not. Literature reports different hypothesis [3,4]. 

Regarding 2D echocardiography evaluation, some studies approached the population of patients with previous cancer using this non-invasive method. In order to evaluate pathological conditions, traditional 2D echocardiography, supported by strain analysis of cardiac chambers, has been applied in a specific trial [5]. In particular we investigated a group of women with previous breast cancer [6], practicing dragon boat sport activity, demonstrating a normal systolic and diastolic function. Furthermore, we studied a group of renal transplants, for the potential cardiotoxicity onset, during a long-term exercise prescription protocol [7].

Strain is a dimensionless quantity that describes the deformation produced by stress and, in addition to ejection fraction, is a non-invasive method for assessment of myocardial function. It represents the percentage change in size compared to original length. The strain rate (SR) is also equivalent to the rate of deformation during time. Left ventricular twist movement is defined as the difference of rotation between the basal segment and the apical one calculated by spatial velocity gradient. All these parameters contribute to identify a normal LV functioning [8]. 

More recently a new system has been created to complete the investigation of the myocardial performance by the vorticity blood flow study. It consists in the hyperdoppler echocardiographic method. This is a sort of eveolution of the echocardiographic investigation. The hyperdoppler method combines the strain analysis with colour image. It has highlighted the role and the importance of the intracardiac vortex and the intracardiac forces. Until now, the evaluation of intracardiac flows was a prerogative of the MRI [9]. The echocardiographic method has been recently validated [10]. The vorticity is a fluid property, based on its local angular velocity, that describes its tendency to rotate. A vortex is a fluid mass of circular or elliptical shape that rotates around a virtual central axis. When the boundaries disappear or expand abruptly (as in the case of systole and diastole), the stress generated by the friction creates a swirling flow of the peripheral layers of fluid that rotate away from the central jet. The flow organization plays a physiological role in the heart’s work and any [10,11] disturbances of intracardiac dynamics, where the laminal flow is substituted by a vorticose one, can be considered to have a negative impact on cardiac function.

We know that fiber orientation and function have been described as a major determinant of blood flow orientation in heart chambers playing an essential role in cardiac performance.

This distribution generates important changes in blood flow direction and magnitude as it passes through the atria and ventricles. Multilayer cardiac magnetic resonance imaging (CMRI) can illustrate asymmetries and changes in the direction of flow through heart chambers redirecting the amount of blood flow to the next cavity. One such change in flow movement is the appearance of eddies, regions of major vorticity.

Eddies are formed as follows: when blood flows through a tubular structure, central fluid layers move faster than those adjacent (due to the frictional containment boundaries) creating eddies.

When the boundaries disappear or expand abruptly (as in the case of systole and diastole), the stress created by the friction generates a swirling tendency of fluid’s peripheral layers that rotate away from the central jet.

Some peculiar geometric and kinetic data of the myocardial chamber are obtained from the Hyperdoppler analysis application. Among them the shape and the movement of the principal vortex in the LV chamber, characterized by deep, length and intensity, are fundamental to distinguish a normal myocardial work from a condition in which heart is involved in a major energy dissipation. Other parameters are kinetic parameters such as vorticity (W), wall shear stress (WSS), relative strength (RS), energy dissipation index (DI), kinetic energy fluctuation (KEF), and vortex fluctuation. These could be considered, especially in sports medicine, important indicators for detecting and monitoring abnormalities in patient’s LV dysfunction. The acquistion is possible following the standard 2D echo imaging, following the American Society of Echocardiography/European Association of Cardiovascular Imaging recommendations [12].

**Figure 1:**
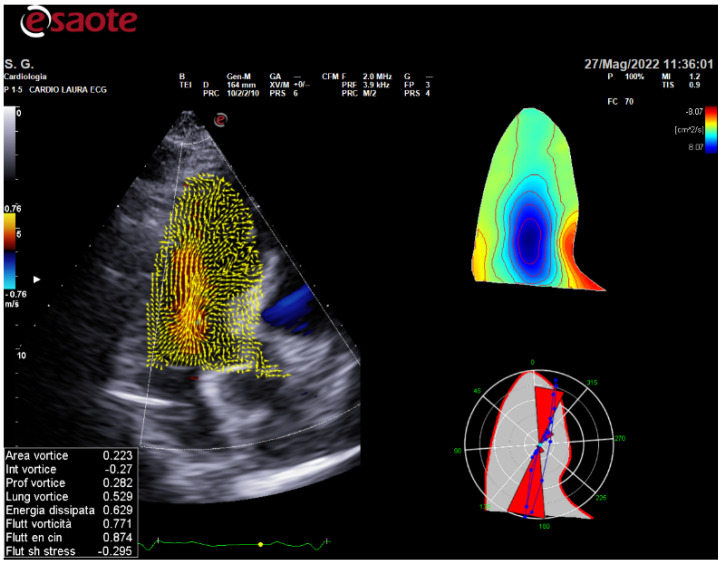
Example of Vortex acquisition and analysis in subjects after renal transplantation and mild hypertension. Caption: the blue colour is the main central flow in the LV chamber. The forces distributions are represented by the red angular flow.

Measures are acquired from 3 cardiac cycles in sinus rhythm or 5 in atrial fibrillation, using Doppler and 2D-mode modalities. Study by hyperdoppler starts with early diastolic filling (transmitral blood flow is directed towards the left ventricle) which leads to formation of a vortex. The presence of vorticities is recognized through flow-based visualization techniques using colordoppler mapping (Figure 1). In first analysis, from the colour point of view, the principal flow direction, and the vector’s forces distribution show that the direction of the flow has a specific length that has some peculiar characteristics in normal if compared to athletes and also to patients. 

In a healthy heart the left ventricle works producing an intraventricular flow pattern to minimize the energy dissipation. Instead in case of abnormal vorticities the transfer in blood flow can increase energy consumption during ejection. The eventual differences can clarify the eventual incorrect situations where the heart woks in a not adequate situation and therefore with high level of energy lost. 

## 2. Conclusions

Strain analisys is recently largely included in the evaluation of athletes especially in case of previous pathological conditions, where the exclusion of cardiotoxicity or reduced contractile function is determinant to plan a physical activity program. The hyperdoppler is a novel ultrasonic biomechanics method based on the integration of both, LV vortex and wall motion, to fully assess and understand the LV structure and function.

The hypothesis is to propose a large use of the hyperdoppler in combination with myocardial deformation study, especially in those subjects where there is a doubt of a cardiomyopathy, despite not evident. The method has a high reproducibility despite not completely compared among the software available. 

Some research proposed the hyperdoppler in the investigation of the dissincronicity of the myocardial function. As recently shown a large clinical application of this method is possible both in healthy people, athletes and pathological clinical situations [9,13]. Considering the wide population investigated in sports medicine, especially subjects with comorbidities, the role of hyperdoppler can find many fields of application, mainly in the follow-up of sports eligibility or exercise prescription. A specific application could be the eventual differences among the different kinds of sports practiced. 

## Data Availability

The study did not report any data.
